# Possible role of *Escherichia coli* in propagation and perpetuation of chronic inflammation in ulcerative colitis

**DOI:** 10.1186/1471-230X-13-61

**Published:** 2013-04-08

**Authors:** Magdalena Pilarczyk-Zurek, Agnieszka Chmielarczyk, Tomasz Gosiewski, Anna Tomusiak, Pawel Adamski, Malgorzata Zwolinska-Wcislo, Tomasz Mach, Piotr B Heczko, Magdalena Strus

**Affiliations:** 1 Department of Microbiology, Jagiellonian University Medical College, Czysta 18 Street, Cracow, 31-121, Poland; 2 Polish Academy of Sciences, Institute of Nature Conservation, 33 Mickiewicza Avenue, Cracow, 31-120, Poland; 3 Department of Gastroenterology, Hepatology and Infectious Diseases, Jagiellonian University Medical College, Śniadeckich 5 Street, Cracow, 31-531, Poland

**Keywords:** Ulcerative colitis, *Escherichia coli*, Iron acquisition

## Abstract

**Background:**

This study investigated a possible role of *Escherichia coli* in propagation and perpetuation of the chronic inflammation in ulcerative colitis (UC). The lesions of UC are located superficially on the rectal and/or colonic mucosa. It is suggested that the commensal bacteria of the digestive tract may play a role in the pathogenesis of UC. Several studies have demonstrated proliferation of *E. coli* in the gut of UC patients. An increase in the number of *E. coli* in the inflamed tissue is most probably related to the abundance of iron ions produced by the bacteria.

**Methods:**

Colon mucosal biopsies were collected from 30 patients with acute-phase UC, both from tissues with inflammatory changes (n = 30) and unchanged tissue with no inflammatory changes (n = 30) from the same patient. Biopsies were also taken from 16 patients with irritable bowel syndrome diarrhea who comprised the control group. Quantitative and qualitative analysis of the biopsy specimens was performed using culture methods and real-time polymerase chain reaction (PCR). Genotyping of the *E. coli* isolates was done using pulsed-field gel electrophoresis. Multiplex PCR was used to compare the *E. coli* strains for the presence of genes responsible for synthesis of iron acquisition proteins: *iroN*, *iutA*, *iha*, *ireA*, *chuA*, and *hlyA.*

**Results:**

We demonstrated that there was a significant increase in the number of *E. coli* at the sites of inflammation in patients with UC compared to the control group (*P* = 0.031). Comparative analysis of the restriction patterns of *E. coli* isolated from inflammatory and unchanged tissues showed that the local inflammatory changes did not promote specific *E. coli* strains. There was a significant difference in the frequency of the *iroN* gene in *E. coli* isolated from patients with UC as compared to the control group.

**Conclusions:**

The increase in the numbers of *E. coli* in the inflammatory tissues is related to the presence of *chuA* and *iutA* genes, which facilitate iron acquisition during chronic intestinal inflammatory processes.

## Background

Ulcerative colitis (UC) is a chronic inflammatory disease and, like Crohn’s disease, belongs to the inflammatory bowel diseases (IBDs) [1,2]. The lesions of UC are located superficially on the rectal and/or colonic mucosa. The clinical course of UC is most commonly composed of exacerbation with periodic remission [3]. Disease activity is assessed based on medical history, as well as endoscopic changes in the large intestine [4].

Initial studies on the microbiology of UC were directed toward determination of a single etiological agent responsible for the development of IBD. Particular attention was paid to bacteria such as *Salmonella*, *Shigella*, *Campylobacter*, *Listeria* and *Mycobacterium* and their possible role in the inflammatory processes in the gastrointestinal (GI) tract [5,6]. None of the studies showed any increase in the populations of the above bacteria in the course of the disease. Currently, it is suggested that commensal bacteria of the digestive tract may play a significant role in the pathogenesis of UC [2,7].

Several studies have demonstrated the proliferation of *Escherichia coli* in the gut of animal models and UC patients [2,8,9]. Studies performed on knockout mouse models (129/SvEv) have shown that chronic gut inflammation in IL-10^−/−^ mice results in a reduction of gut microbiota diversity and a strong increase in intestinal *E. coli*[10].

The increase in the number of *E. coli* in the inflammatory tissue is most probably related to the abundance of iron ions available for siderophores produced by the bacteria [11]. Therefore, the significant increase in *E. coli*, but not their high virulence, may have an influence on the inflammatory process in the GI tract.

For *E. coli* that colonizes the human colon, it is important to have sufficient iron in the cells. In the process of acquiring iron ions, it is necessary for the cell to be able to produce transmembrane proteins that play a receptor role for siderophores that chelate iron ions. The receptor protein for hemin, which allows bacteria to use iron from heme, is coded by the *chuA* gene; synthesis of the receptor protein for phenol-derived siderophores is dependent on the *iron* gene; the protein that is an analog of the adhesion-forming part of the outer membrane is coded by the *iha* gene; and outer membrane proteins involved in binding other siderophores are coded by the *iutA* and *ireA* genes. Among *E. coli* strains isolated from patients suffering from diseases of the GI tract, strains that have the ability to lyse erythrocytes have been identified, with hemolysin α being the best characterized. Biosynthesis of the active form of the enzyme requires *hlyC*, *hlyA*, *hlyB* and *hlyD* genes, post-translational modification, and secretion by protein translocators [12,13].

There are reports that some *E. coli* genotypes are more likely associated with IBD than others [14]. In particular, *E. coli* strains belonging to phylogenetic groups associated with virulence genes and the ability to adhere to and invade host cells are more frequently isolated from IBD patients than from control subjects [9,15]. So far there has been no universal agreement as to which specific features are responsible for their dominance.

Studies on animal models [10] have not shown a clear-cut correlation between increases in *E. coli* cell numbers and the severity of inflammation, which supports the assumption that high *E. coli* numbers are a consequence rather than a cause of disease. Furthermore, it has been shown that nonpathogenic *E. coli* strains even accelerate remission [16].

The aims of the present study were: (1) quantitative assessment of most important bacterial groups, with special emphasis on *E. coli* in patients with the active phase of UC, in sites of inflammatory changes and unchanged mucosa, as well as in the control group, based on culture methods and real-time polymerase chain reaction (PCR); (2) comparative genotyping of *E. coli* strains isolated from the same patients with active-phase UC from inflammatory and unchanged mucosa; and (3) comparison of the frequency of genes present, which are responsible for production of factors that facilitate iron ion acquisition (*iroN*, *iutA*, *iha*, *ireA*, *chuA* and *hlyA)*.

## Methods

### Study subjects

The study material consisted of biopsies collected during colonoscopy from the colon mucosa of 30 patients with a diagnosis of acute-phase UC and from 16 patients with irritable bowel syndrome diarrhea who comprised the control group. The study was performed in the Clinic of Gastroenterology and Hepatology of the University Hospital in Cracow and in the Microbial Ecology Laboratory of the Chair of Microbiology, Jagiellonian University in 2008–2011, after approval by the Jagiellonian University Bioethical Committee (no. KBET/75/B from 15.11.2007). Informed consent was obtained from all patients participating in the study.

The average age of patients with UC was 42 ± 11 years, and the average disease duration was 5 years (1–23 years), with an average exacerbation rate of 1.5/year. Activity of UC and exacerbation of endoscopic changes in the colon were based on the Mayo Clinic Disease Activity Index [4]. All subjects underwent the same type of preparation prior to colonoscopy, with oral sodium phosphate at a dose of 0.6–0.8 ml/kg (up to 45 ml) and bowel cleansing, consisting of four saline enemas. During colonoscopy, patients received intravenous sedation or general anesthesia, as required. Two biopsy specimens were collected from each patient with UC: one from the colonic mucosa with inflammatory changes (Sample A, n = 30); and the other from mucosa with no inflammatory signs (Sample B, n = 30). Inflammatory tissue was characterized during colonoscopy by disappearance of the vascular network, fragile mucosa with bleeding on contact, ulcerations, erosions, and sometimes pseudopolyps. The unaffected tissue showed normal mucosa with visible blood vessels, with no redness or mucosal depletion in the form of ulcers or erosions. In patients from the control group, no inflammatory changes were noted in the colon mucosa and only single biopsies were collected (Sample C, n = 16). All tissues were subjected to histopathological assessment according to the Geboes scale [17]. The unaffected tissue from UC patients and mucosa from the control group received zero points according to the scale. The exclusion criteria were: diabetes, autoimmune disorders, severe systemic diseases, alcohol abuse, cow’s milk allergy, and nonsteroidal anti-inflammatory drugs intake. All patients enrolled in the study received no antibiotics for at least 3 months before colonoscopy. Patients with acute-phase UC received standard treatment with 3 g/day mesalamine.

### Qualitative and quantitative identification of bacterial species

#### Culture methods

The collected biopsies were suspended in Schaedler broth (SAB; Difco, USA) with 10% glycerol and stored at −20°C for up to 1 week. The samples were transported to the microbiology laboratory on dry ice. The frozen samples were thawed, weighed, homogenized in 1 ml SAB, and quantitatively analyzed for the main bacterial constituents by culture on differential media in aerobic and anaerobic conditions [18]. All these manipulations were done aseptically in an anaerobic chamber (MACS; Don Whitley, Shipley, Yorks, UK) in an atmosphere of N (85%) + H_2_ (10%) + CO_2_ (5%). Homogenized samples were serially diluted with SAB and 100-μl aliquots were plated on the following media: McConkey Agar (Oxoid, Basingstoke, Hants, UK) for *Enterobacteriaceae*; Columbia Blood Agar (Difco) with 5% sheep blood for streptococci; BBL Enterococcosel Agar (BD, Franklin Lakes, NJ, USA) for enterococci; MRS Agar (Oxoid) for lactobacilli and other lactic acid bacteria; BL Agar for bifidobacteria; and Wilkins–Chalgren Agar Base with supplements for *Bacteroides*[19]. The morphology of the colonies was analyzed under a magnifying glass and several colonies (7–10) of each morphological type were subcultured on appropriate aerobic and anaerobic media and Gram stained. After further incubation and culture purity checks, phenotypic identification was performed using commercial identification systems (API 20E, API 20A, API Staph, and API Strept; bioMerieux, Marcy l’Etoile, France; BBL Crystal ID System; BD). To verify speciation, all Gram-negative rods tested with API 20E were additionally analyzed with PCR with species-specific primers for *E. coli*[20]. The numbers of colonies of the bacterial groups and the sum of the cultured bacteria in the weighed tissue samples were converted into 1 g of the mass to make the quantitative comparisons among the patients.

### Real-time PCR

Real-time PCR was used to verify the results obtained with culture methods. DNA extraction from all tissue samples was performed using the Genomic Mini Isolation Kit (A&A Biotechnology, Poland), according to the manufacturer’s recommendations, with our own modification. After lysis of the bacterial cells with lysozyme (1 mg/ml) and lysostaphin (0.1 mg/ml), samples were incubated at 37°C for 20 min. Next, 200 μl 75 mM NaOH (50 mM) was added and samples were incubated at 95°C for 10 min. After incubation, probes were microcentrifuged (12 000 rpm, 10 min), supernatants were removed, and pelets were resuspended in 500 μl buffer suplemented with β-mercaptoethanol (Sigma). For each sample, lyticase was added (0.1 mg/ml). Probes were incubated at 37°C for at least 30 min and microcentrifuged (12 000 rpm, 10 min). The next steps of DNA extraction were carried out according to A&A Biotechnology’s procedure.

**Figure 1:**
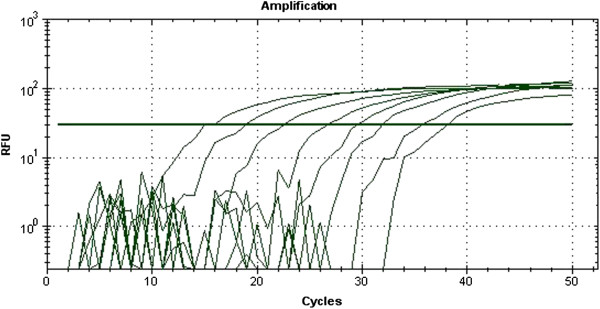
**Relative fluorescence is the increase in reporter dye intensity relative to the passive internal reference dye.** The amount of *E. coli* DNA in each sample is shown in the key. The threshold fluorescence, or level at which the threshold cycle was determined, is shown.

**Figure 2:**
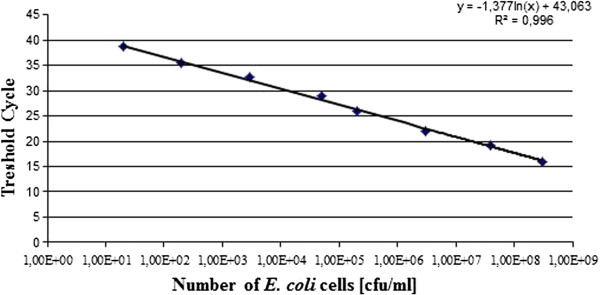
**Standard curve was generated from the amplification plot in Figure**1**.** y= −1.377 ln(x) + 43.063; correlation coefficient =0.996. Threshold cycle was the cycle number when the threshold fluorescence was reached.

To detect specific DNA sequences after extraction, fluorescently labeled probes and pairs of specific primers were used (Table 1). *E. coli* in the corresponding tissue samples was quantified by Real-Time PCR, as described previously [21]. A standard curve was prepared. DNA from given numbers of *E. coli* was added in serial dilutions from 10^1^ to 10^8^ cells to a series of PCRs. The reactions were carried out in a BioRad thermocycler, and the fluorescence was monitored throughout the reaction. The results are shown in Figure 1. A standard curve from these data is shown in Figure 2. Detection and quantitation were linear over the range of DNA concentrations examined. To determine the number of *E. coli* cells, the fluorescent signals detected from two serial dilutions in the linear range of the assay were averaged and compared to a standard curve (Figure 2).

**Table 1:** Primers and probes used in this study

**Gene**	**Product size [bp]**	**Oligonucleotide sequence**
*hlyA*	1177	GTA TAC ACA AAA GAA GGA AGC
		ACA GAA TCG TCA GCA TCA GC
*iroN*	667	AAG TCA AAG CAG GGG TTG CCC G
		GACGCCGACATTAAGACGCAG
*iutA*	302	GGC TGG ACA TCA TGG GAA CTG G
		CGT CGG GAA CGG GTA GAA TCG
*iha*	829	CTG GCG GAG GCT CTG AGA TCA
		TCC TTA AGC TCC CGC GGC TGA
*ireA*	254	GAT GAC TCA GCC ACG GGT AA
		CCA GGA CTCA CCT CAC GAA T
*chuA*	279	GAC GAA CCA ACG GTC AGG AT
		TGC CGC CAG TAC CAA AGA CA
Primers and probe specific for *E. coli* strains (Real-time PCR)		
16S rRNA	204	GGG AGT AAA GTT AAT ACC TTT GC
		CTC AAG CTT GCC AGT ATC AG
Probe		HEX- CGC GAT CAC TCC GTG CCA GCA GCC GCG GAT CGC G -BHQ1

### Genotyping of *E. coli* using pulsed-field gel electrophoresis (PFGE)

To genotype and compare *E. coli* strains isolated from the inflammatory and unchanged tissues from the same patient, we performed comparative analysis using PFGE. Preparation of the agarose blocks, conditions of enzymatic digestion with *Xba*I and electrophoretic parameters were carried out according to the international PulseNet CDC (PulseNet 2002) guidelines [22]. The reference strain − *Salmonella Braenderup* H9812 – was obtained by courtesy of the staff of the National Institute of Public Health – National Institute of Hygiene in Warsaw. PFGE banding patterns were analyzed with the Molecular Analyst software (BioRad) using Dice coefficient and UPGMA (unweighted pair group method with arithmetic averages) algorithm.

### Multiplex PCR

When choosing genes for PCR, we concentrated on the property of *E. coli* to acquire iron ions, that is: *iroN*, *iutA*, *ireA* and *chuA* genes coding receptor proteins for siderophores; *iha* coding the protein analog of adhesin; and *hlyA* gene responsible for hemolysin α synthesis, the enzyme that degrades erythrocytes and liberates hemin. Multiplex PCR was performed according to the method of Zhao et al. [23] and PCR following the procedure of Aranda et al. [24]. Primer sequences and the size of the amplification products are shown in Table 1.

### Detection of hemolytic activity

All isolated *E. coli* strains were tested for the production of hemolysin on blood agar plates, prepared with defibrinated sheep blood washed three times and added to Trypticase Soy Agar (Columbia Lab Agar; Biocorp) at a final concentration of 5%. Production of hemolysis was characterized by the formation of a clear halo around bacterial colonies after overnight incubation at 37°C. The absence of hemolytic activity characterized nonhemolytic *E. coli* strains.

### Evaluation of relations between numbers of *E. coli* expressing *iroN, iutA, iha, ireA, chuA* and *hlyA* and their isolation sites

To obtain a general model for the factors influencing the number of bacteria, a multivariate statistical model was constructed. The model was based on the general linear model assumption. Bacterial abundance was assumed to be the continuous variable with exponential distribution dependent on the set of categorical predictors: absence of the analyzed genes (*iroN*, *iutA*, *iha*, *ireA*, *chuA* and *hlyA)*; group of patients; and the site of material origin.

### Statistical analysis

Statistical analysis was done using Microsoft Access and Statistica software packages. Likelihood ratio and *χ*^2^ tests were used. *P* < 0.05 was considered significant. The model used to evaluate the relations between the numbers of *E. coli* expressing the different genes and their isolation sites significantly explained the statistical relations (*χ*^2^ = 65,2614, df = 8, *P* < 0.0001).

## Results

### Qualitative and quantitative assessment of bacterial species based on culture methods

Based on classical culture methods, we showed that there were significantly more lactobacilli in the inflammatory tissues (Sample A) compared to the control group (*P* = 0.044), and in the noninflammatory mucosa (Sample B) compared to the control group (*P* = 0.041). There were no significant differences in the numbers of *Enterococcus*, *Streptococcus* and *Bifidobacterium* isolated from inflammatory and unchanged tissues in patients with UC compared with the control group. For *Enterobacteriacae* with special consideration of *E. coli*, no quantitative differences were observed between the UC patients and control group. The results are shown in Table 2.

**Table 2:** Numbers of bacteria isolated from inflammatory and unchanged colonic mucosa in patients with UC

**Bacteria**	**Average bacterial no. [c.f.u/g] isolated from patients in active phase of UC, n=30**		**Control group, (sample C, n=16)**
	**Inflammatorily changed site biopsy (sample A, n=30)**	**Unchanged mucosal biopsy (sample B, n=30)**	
Sume of cultured bacteria	3.1·10^7^±1.6·10^7^	6.4·10^7^±4.8·10^7^	7.6·10^7^±6.1·10^7^
*Enterobacteriaceaae*	5.0·10^6^±1.8·10^6^	2.1·10^7^±1.1·10^7^	1.5·10^7^±8.1·10^6^
Escherichia coli ***	3.3·10^6^±3.1·10^6^	1.3·10^6^±1.1·10^6^	1.6·10^6^±1.2·10^6^
*Enterococcus*	8.9·10^6^±6.3·10^6^	9.5·10^6^±4.5·10^6^	4.5·10^6^±3.4·10^6^
*Streptococcus*	3.2·10^6^±2.0·10^6^	9.1·10^6^±6.8·10^6^	7.1·10^6^±5.9·10^6^
*Lactobacillus*	5.2·10^6^ ±3.0·10^6^*	8.5·10^6^±5.0·10^6^ **	7.6·10^5^±4.4·10^5^
*Bifidobacterium*	5.1·10^6^±3.5·10^6^	2.1·10^6^±1.1·10^6^	2.7·10^5^±1.2·10^5^

Results based on culture methods.

* Numbers significantly different from the control group (*P = 0.044; **P = 0.041).

*** *E. coli* was found in only 18 patients with active-phase UC and 12 patients from the control group.

Based on culture methods, 52 *E. coli* strains were isolated from 18 patients with acute-phase UC: 25 strains from Samples A, 27 strains from Samples B, and 18 strains from 12 patients in the control group. In total, 70 *E. coli* strains were investigated further.

### Quantitative assessment of *E. coli* based on real-time PCR

The bacterial DNA sequences isolated from all 76 tissue samples were analyzed. The presence of *E. coli* DNA was confirmed in 48 tissue samples (including 36 samples from 18 patients with acute-phase UC: Sample A, n = 18; Sample B, n = 18; and from 12 patients in the control group, Sample C, n = 12. There was a significant increase in the *E. coli* populations in the inflammatory tissues (Sample A) from patients with UC compared with the control group (*P* = 0.031), but not in the non-inflammatory tissues (Sample B). The results are shown in Table 3.

**Table 3:** Numbers of bacteria isolated from inflammatory and unchanged colonic mucosa in patients with UC

**Bacteria**	**Average bacterial no. [c.f.u/g] isolated from patients in active phase of UC, n=30**		**Control group, (sample C, n=16)**
	**Inflammatorily changed site biopsy (sample A, n=30)**	**Unchanged mucosal biopsy (sample B, n=30)**	
Escherichia coli ***	3.95·10^13^±3.9·10^13^*	2.21·10^10^±3.1·10^10^	9.17·10^10^±6.5·10^10^

Results based on Real-time PCR.

* Numbers significantly different from the control group (*P = 0.031).

*** *E. coli* was found in only 18 patients with active-phase UC and 12 patients in the control group.

### PGFE genotyping of *E. coli* isolated from inflammatory and unaffected tissues in patients with active-phase UC

**Figure 3:**
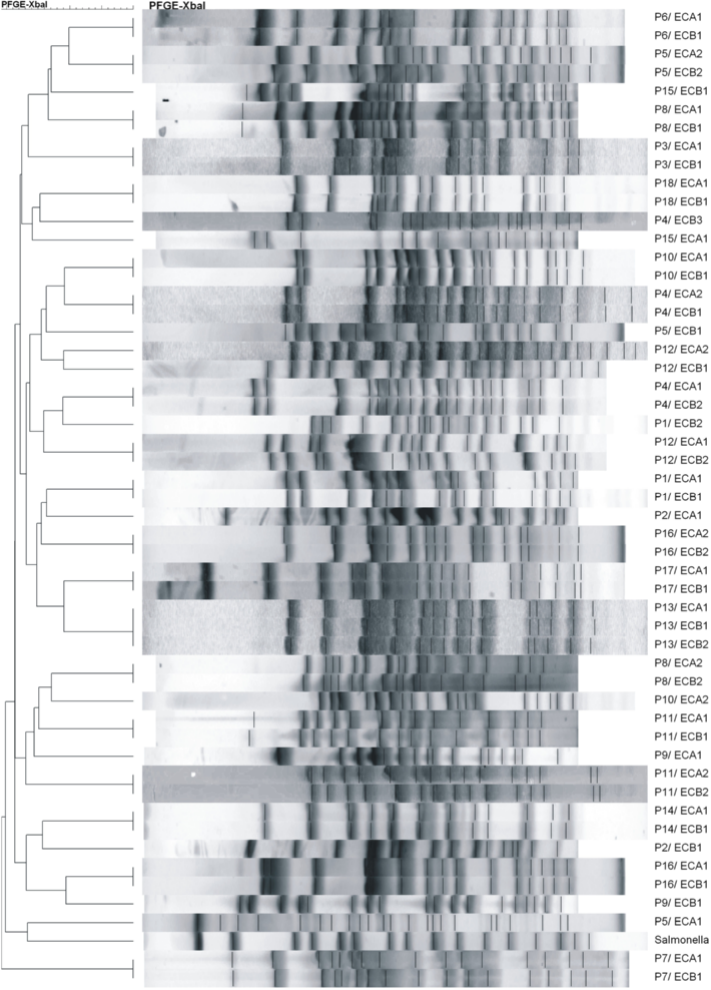
**Comparison of restriction patterns of *E. coli *isolated from patients with active-phase UC.** Results based on PFGE. P1/ECA1- P18/ECB1 - strains isolated from UC patients (A-inflammatorily changed place, B – unchanged mucosa, reference strain - *Salmonella Braenderup* H9812.

Isolated *E. coli* strains were characterized by high genetic variability. The analyses were done for all patients with confirmed *E. coli* strains (n = 18), including 52 strains originating from Samples A (25 strains) and Samples B (27 strains). Among the 52 strains, 32 different pulsotypes were noted. In 15 patients (83%), the *E. coli* strains isolated from inflammatory and unchanged tissues had identical restriction profiles. Only three patients had genetically different strains isolated from Sample A vs. Sample B. Additionally, in eight patients there was more than one *E. coli* strain isolated from the given site with a unique genetic profile. The results are shown in Figure 3.

### Frequency of genes responsible for synthesis of proteins for acquisition of iron ions

**Figure 4:**
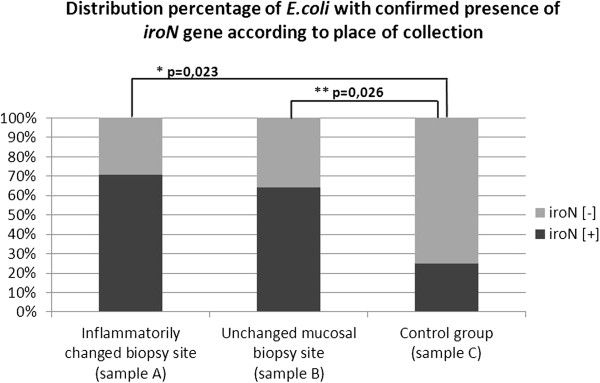
**Distribution of *E. coli *with confirmed *iroN *gene.** Specimens isolated from colon mucosa with inflammatory changes and unchanged mucosa from patients with active-phase UC compared with the control group.

Based on PCR, we tested for the frequency of genes in the genomes of 70*E. coli* strains. Analyzing the frequency of the genes responsible for synthesis of proteins for acquisition of iron ions (*iroN*, *iutA*, *iha*, *ireA*, *chuA* and *hlyA*), we showed significant differences in relation to the frequency of *iroN* gene coding the protein responsible for binding siderophores. The *iroN* gene was confirmed in 17 of 25 *E. coli* strains isolated from Samples A (inflammatory tissue); in 18 of 27 strains isolated from Samples B (unchanged tissue); as well as in six of 18 strains isolated from patients in the control group. A higher number of *E. coli* isolates from UC patients had the *iroN* gene in comparison with the strains isolated from the control group. No significant differences were shown for the frequency of the *iroN* gene in the strains from Samples A and B from the same patient. The results are shown in Table 4 and Figure 4.

**Table 4:** Frequency of genes responsible for synthesis of proteins for acquisition of iron ions

**Number**	**Hemolysis - phenotypic**	**Gene presence**					
		***hlyA***	***iha***	***iroN***	***chuA***	***iutA***	***ireA***
**P1/ECA1**	[−]	[−]	[−]	[−]	[−]	[−]	[−]
**P1/ECB1**	[−]	[−]	[−]	[−]	[−]	[−]	[−]
**P1/ECB2**	[+]	[+]	[−]	[+]	[+]	[−]	[−]
**P2/ECA1**	[−]	[−]	[−]	[+]	[−]	[+]	[+]
**P2/ECB1**	[−]	[−]	[−]	[−]	[−]	[−]	[−]
**P3/ECA1**	[−]	[−]	[−]	[+]	[+]	[−]	[−]
**P3/ECB1**	[−]	[−]	[−]	[+]	[+]	[+]	[−]
**P4/ECA1**	[−]	[−]	[−]	[+]	[−]	[−]	[−]
**P4/ECA2**	[−]	[−]	[−]	[+]	[−]	[−]	[−]
**P4/ECB1**	[−]	[−]	[−]	[+]	[−]	[−]	[−]
**P4/ECB2**	[−]	[−]	[−]	[+]	[−]	[−]	[−]
**P4/ECB3**	[−]	[−]	[−]	[+]	[−]	[+]	[−]
**P5/ECA1**	[−]	[−]	[−]	[+]	[+]	[−]	[−]
**P5/ECA2**	[−]	[−]	[−]	[−]	[−]	[−]	[−]
**P5/ECB1**	[−]	[−]	[−]	[+]	[+]	[−]	[−]
**P5/ECB2**	[−]	[−]	[−]	[−]	[−]	[+]	[−]
**P6/ECA1**	[−]	[−]	[−]	[−]	[−]	[−]	[−]
**P6/ECB1**	[−]	[−]	[−]	[−]	[−]	[−]	[−]
**P7/ECA1**	[−]	[−]	[−]	[+]	[−]	[+]	[−]
**P7/ECB1**	[−]	[−]	[−]	[+]	[−]	[+]	[−]
**P8/ECA1**	[+]	[+]	[−]	[+]	[+]	[−]	[−]
**P8/ECA2**	[−]	[−]	[+]	[−]	[+]	[+]	[−]
**P8/ECB1**	[+]	[+]	[−]	[+]	[+]	[−]	[−]
**P8/ECB2**	[−]	[−]	[+]	[−]	[+]	[+]	[−]
**P9/ECA1**	[−]	[−]	[−]	[+]	[−]	[+]	[−]
**P9/ECB1**	[−]	[−]	[+]	[−]	[+]	[+]	[−]
**P10/ECA1**	[−]	[−]	[−]	[+]	[−]	[−]	[−]
**P10/ECA2**	[−]	[−]	[−]	[+]	[−]	[+]	[+]
**P10/ECB1**	[−]	[−]	[−]	[+]	[−]	[+]	[+]
**P11/ECA1**	[−]	[−]	[−]	[+]	[−]	[+]	[−]
**P11/ECA2**	[−]	[−]	[−]	[+]	[+]	[+]	[−]
**P11/ECB1**	[−]	[−]	[−]	[+]	[−]	[+]	[−]
**P11/ECB2**	[−]	[−]	[−]	[+]	[+]	[+]	[−]
**P12/ECA1**	[−]	[−]	[−]	[+]	[+]	[+]	[−]
**P12/ECA2**	[+]	[+]	[−]	[−]	[−]	[−]	[−]
**P12/ECB1**	[+]	[+]	[−]	[+]	[+]	[+]	[−]
**P12/ECB2**	[−]	[−]	[−]	[+]	[+]	[−]	[−]
**P13/ECA1**	[+]	[+]	[+]	[−]	[+]	[+]	[−]
**P13/ECB1**	[+]	[+]	[+]	[−]	[+]	[+]	[−]
**P13/ECB2**	[+]	[+]	[+]	[−]	[+]	[+]	[−]
**P14/ECA1**	[−]	[−]	[−]	[+]	[−]	[−]	[−]
**P14/ECB1**	[−]	[−]	[−]	[+]	[−]	[−]	[−]
**P15/ECA1**	[−]	[−]	[−]	[−]	[−]	[−]	[−]
**P15/ECB1**	[−]	[−]	[−]	[−]	[−]	[−]	[−]
**P16/ECA1**	[−]	[−]	[−]	[−]	[−]	[−]	[−]
**P16/ECA2**	[−]	[−]	[−]	[+]	[−]	[+]	[−]
**P16/ECB1**	[−]	[−]	[−]	[+]	[−]	[+]	[−]
**P16/ECB2**	[−]	[−]	[−]	[+]	[−]	[+]	[−]
**P17/ECA1**	[−]	[−]	[−]	[+]	[+]	[+]	[−]
**P17/ECB1**	[−]	[−]	[−]	[+]	[+]	[+]	[−]
**P18/ECA1**	[−]	[−]	[−]	[+]	[−]	[−]	[−]
**P18/ECB1**	[−]	[−]	[−]	[+]	[−]	[−]	[−]
**P1/ECC1**	[−]	[−]	[−]	[+]	[−]	[+]	[−]
**P1/ECC2**	[+]	[+]	[+]	[+]	[+]	[+]	[−]
**P2/ECC1**	[−]	[−]	[+]	[−]	[+]	[+]	[−]
**P3/ECC1**	[−]	[−]	[−]	[+]	[+]	[+]	[−]
**P3/ECC2**	[−]	[−]	[−]	[+]	[+]	[−]	[−]
**P4/ECC1**	[−]	[−]	[−]	[−]	[+]	[−]	[−]
**P5/ECC1**	[−]	[−]	[−]	[−]	[+]	[−]	[−]
**P5/ECC2**	[−]	[−]	[−]	[−]	[−]	[−]	[−]
**P6/ECC1**	[−]	[−]	[−]	[−]	[−]	[+]	[−]
**P7/ECC1**	[−]	[−]	[+]	[−]	[+]	[−]	[−]
**P8/ECC1**	[+]	[+]	[−]	[−]	[+]	[+]	[−]
**P8/ECC2**	[−]	[−]	[+]	[−]	[+]	[−]	[−]
**P9/ECC1**	[−]	[−]	[−]	[+]	[−]	[−]	[−]
**P10/ECC1**	[−]	[−]	[−]	[−]	[−]	[+]	[−]
**P10/ECC2**	[−]	[−]	[+]	[−]	[+]	[+]	[−]
**P11/ECC1**	[−]	[−]	[−]	[+]	[+]	[+]	[−]
**P12/ECC1**	[−]	[−]	[−]	[−]	[−]	[−]	[−]
**P12/ECC2**	[−]	[−]	[−]	[−]	[+]	[−]	[−]

Genes studied: *iroN*, *iutA*, *iha*, *ireA*, *chuA* and *hlyA.*

+ positive result; – : negative result.

P1/ECA1–P18/ECB1: strains isolated from UC patients (A: inflammatory tissues; B: unchanged mucosa); P1/ECC1–P12/ECC2: strains isolated from the control group.

### Detection of hemolytic activity

**Figure 5:**
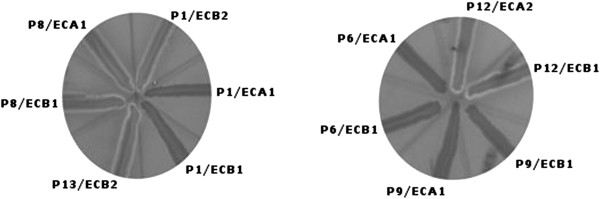
**α-Hemolysis visible on blood agar.***E. coli*: P1/ECB2, P8/ECA1, P8/ECB1, P12/ECA2, P12/ECB1, P13/ECB2 – visible type alpha hemolysis, strains: P1/ECA1, P1/ECB1, P6/ECA1, P6/ECB1, P9/ECA1, P9/ECB1 – no alpha hemolysis.

When analyzing the ability of *E. coli* to lyse red blood cells, 10 of 70 strains cultured on blood agar showed phenotypic hemolytic characteristics. α-Hemolysis in selected strains is shown in Figure 5. Furthermore, 70 *E. coli* strains were tested with PCR to confirm the presence of the *hlyA* gene, which is responsible for biosynthesis of α-hemolysin. In 10 strains that showed phenotypic α hemolysis, the presence of *hlyA* was confirmed. These strains were isolated with the same frequency from inflammatory and unchanged tissues.

### Relations between numbers of *E. coli* expressing *iroN*, *iutA*, *iha*, *ireA*, *chuA* and *hlyA* and their isolation sites

Analysis showed that three predictors significantly influenced the dependent variable: presence of genes *chuA* and *iutA* as well as place of origin (inflammatory colonic mucosa, Sample A and unchanged mucosa, Sample B). Presence of *chuA* and *iutA* genes in *E. coli* correlated with the increase in bacteria assessed using real-time PCR in the inflammatory tissues where there was more free iron available.

## Discussion

Currently, a lot of attention is given to the role of commensal bacteria in the human GI tract, the numbers of which change in relation to persistent intestinal inflammatory processes [2,7-9,25].

Mylonaki et al. have shown a significant increase in the population of *E. coli* from colonic mucosa biopsies collected from UC patients compared with those in remission and the control group [26]. Kleessen at al. [27] have analyzed quantitative changes in bacterial populations in patients with UC versus a control group, and demonstrated an increase in the populations of *Enterobacteriacae*, including *E. coli.* In both studies, the quantitative analysis was based on fluorescence *in situ* hybridization (FISH).

We confirmed the relationship between an increase in the population of Gram-negative rods (*E. coli*) and exacerbation of clinical symptoms of UC. Based on real-time PCR, we demonstrated a significant increase in *E. coli* in inflammatory tissues in patients with UC compared with the control group.

In most recent studies, microorganisms in tissue samples from patients with different IBDs have been assessed using molecular methods (e.g. PCR, real-time PCR, and FISH), which allow one to detect even trace amounts of microbial DNA. These methods are especially useful for quantitative analysis of tissue samples from patients with chronic inflammatory diseases. In places where there are ongoing biochemical reactions related to the inflammatory process, reactive oxygen species (ROS) are abundant, which has a direct influence causing a decrease in viability of bacterial cells [28]. These reactions are very dynamic, leading to sudden changes in bacterial populations, depending on the presence of multiple proinflammatory factors. Using methods with the highest sensitivity (real time PCR) play a key role in such a situation. The real-time PCR system of Ott et al. [29] provides an accurate and stable method to measure bacterial concentrations in clinical samples, but validation of the results obtained by real-time PCR with traditional bacterial culture methods is difficult to perform and can generate mean differences [29]. Therefore qualitative and quantitative assessment based solely on classical culture methods during exacerbation of the inflammatory process in the GI tract seems insufficient. This may also explain the differences observed in our studies.

The results presented above from our as well as other studies point to a relation between the quantitative changes in *E. coli* and the course of UC. Wohlgemuth et al. used mouse models (129/SvEv) to try to explain quantitative and qualitative changes within the commensal microbiota during inflammation of the GI tract. Their study showed a decrease in variability of microorganisms and a strong increase in *E. coli* numbers during the development of inflammation. At the same time, they found that there was a lack of evidence directly pointing to the increased numbers of *E. coli* as the reason for exacerbation of the inflammatory process. In fact, their conclusion was that the conditions that accompany acute inflammation, favor *E. coli* proliferation [10].

The increase in *E. coli* populations probably depends on many adaptive factors, for example, biofilm formation, synthesis of enzymes able to catalyze breakdown of ROS, use of supportive mechanisms allowing absorption of iron ions from the environment, and ability to acquire iron ions by using hemoglobin from lysed erythrocytes [10,12].

For bacteria colonizing the human GI tract, especially *Enterobacteriacae*, to obtain enough iron for their cells is difficult, owing to the fact that iron is contained in complexes with host proteins (e.g. hemoglobin, transferrin, and lactoferrin) [30]. *E. coli* has the ability to lyse red blood cells, degrade hemoglobin, and acquire iron as a result of the ChuA receptor on the outer membrane [12]. In our study, we noted a significant increase in *E. coli* that possessed the *chuA* gene, which codes for a receptor for hemin from lysed erythrocytes. This was confirmed in patients with UC in an inflammatory tissue. Similarly, a significant relation was confirmed for *E. coli* bearing the *iutA* gene, which is responsible for coding a receptor that allows absorption of iron chelated by one of the siderophores (aerobactin) [30]. The presence of the *chuA* and *iutA* genes in *E. coli* correlates with the increased numbers of bacteria assessed using real-time PCR in inflammatory tissues where more free iron is available. Additionally, we observed significant differences in the presence of *iroN* gene in *E. coli*. The *iroN* gene encodes a receptor that is responsible for identifying and binding siderophores (glucosylated enterobactins), expression of which depends on the presence of iron ions in the environment [30]. The presence of *iroN* gene was confirmed in 70% and 65% of *E. coli* strains isolated from inflammatory and unchanged tissues in UC patients, respectively, compared with 25% of strains from the control group. Based on our present and other previous studies [10], we can conclude that increased availability of iron ions in the GI tract of patients with UC is a significant factor related to the quantitative increase in *E. coli*.

Our study not only assessed *E. coli* quantitatively in UC and control group patients, but also compared the genetic profiles of the strains based on PFGE. Comparative analysis of the restriction patterns confirmed high variability among the *E. coli* strains. It seems that the focal inflammatory state does not favor adhesion of a specific type of *E. coli*. In 83% of cases (15/18 patients), genetically identical *E. coli* were isolated from inflammatory lesions compared with unchanged tissue. Different profiles of *E. coli* collected from the two types of tissues were demonstrated in only three patients. There were no similarities between strains isolated from different patients. Similar confirmatory results were obtained by Thomazini et al. [31], who used ERIC2-PCR (Enterobacterial Repetitive Intergenic Consensus-PCR) in their analysis of 131 *E. coli* strains isolated from patients with IBD (including UC) and a control group. They demonstrated unequivocally that there was no specific strain or group of strains of *E. coli* related to UC or Crohn’s disease, or in the control group. Sepehri et al. [2] have compared *E. coli* strains from patients with IBD (including UC) and a control group, using MLST (Multilocus Sequence Typing). Three main groups of *E. coli* were drawn but no relation was found for the strains and disease entity.

It is worth mentioning that so far there is no proof of a single *E. coli* strain participating in the etiopathogenesis of UC [16]. It is more often considered that there is an increase in *E. coli* populations that can bind free iron ions by siderophores [12] and store iron intracellularly [32], which allows them to inhibit the Fenton reaction in the intestine, by eliminating iron ions and prevention of ROS formation, and at the same time reducing damage to the host tissues [28]. Furthermore, considering the role of *E. coli* as an anti-inflammatory and facilitating remission, there have been some studies on *E. coli* Nissle as a probiotic [33].

## Abbreviations

GI: Gastrointestinal tract; IBD: Inflammatory bowel diseases; PCR: Polymerase chain reaction; PFGE: Pulsed field gel electrophoresis; ROS: Reactive oxygen species UC ulcerative colitis.

## Competing interests

The authors declare that they have no competing interests.

## Authors’ contributions

MPZ, AC, TG and AT carried out the diagnosis of bacterial strains and molecular genetic studies; PA participated in statistical analysis of the data; MZW and TM carried out the clinical research; MS and PBH contributed to conception and design of the project and were involved in data analysis and interpretation of results. All authors read and approved the final manuscript.

## Pre-publication history

The pre-publication history for this paper can be accessed here:

http://www.biomedcentral.com/1471-230X/13/61/prepub
